# Engineered antibodies: new possibilities for brain PET?

**DOI:** 10.1007/s00259-019-04426-0

**Published:** 2019-07-24

**Authors:** Dag Sehlin, Stina Syvänen, on behalf of the MINC faculty, Bénédicte Ballanger, Henryk Barthel, Gérard N Bischof, Delphine Boche, Hennig Boecker, Karl Peter Bohn, Per Borghammer, Donna Cross, Donato Di Monte, Alexander Drzezga, Heike Endepols, Kathrin Giehl, Michel Goedert, Jochen Hammes, Oskar Hansson, Karl Herholz, Günter Höglinger, Merle Hönig, Frank Jessen, Thomas Klockgether, Pierre Lafaye, Adriaan Lammerstma, Eckhard Mandelkow, Eva-Maria Mandelkow, Andreas Maurer, Brit Mollenhauer, Bernd Neumaier, Agneta Nordberg, Özgur Onur, Kathrin Reetz, Elena Rodriguez-Vietez, Axel Rominger, James Rowe, Osama Sabri, Anja Schneider, Antonio Strafella, Stina Syvänen, Thilo van Eimeren, Neil Vasdev, Victor Villemagne, Dieter Willbold

**Affiliations:** Department of Public Health and Caring Sciences/Geriatrics, Uppsala University, Rudbeck Laboratory, 75185 Uppsala, Sweden

**Keywords:** Transferrin receptor 1 (TfR1)-mediated transcytosis, Alzheimer’s disease (AD), Amyloid-β (Aβ), Antibody, Blood–brain barrier (BBB), Positron emission tomography (PET)

## Abstract

Almost 50 million people worldwide are affected by Alzheimer’s disease (AD), the most common neurodegenerative disorder. Development of disease-modifying therapies would benefit from reliable, non-invasive positron emission tomography (PET) biomarkers for early diagnosis, monitoring of disease progression, and assessment of therapeutic effects. Traditionally, PET ligands have been based on small molecules that, with the right properties, can penetrate the blood–brain barrier (BBB) and visualize targets in the brain. Recently a new class of PET ligands based on antibodies have emerged, mainly in applications related to cancer. While antibodies have advantages such as high specificity and affinity, their passage across the BBB is limited. Thus, to be used as brain PET ligands, antibodies need to be modified for active transport into the brain. Here, we review the development of radioligands based on antibodies for visualization of intrabrain targets. We focus on antibodies modified into a bispecific format, with the capacity to undergo transferrin receptor 1 (TfR1)-mediated transcytosis to enter the brain and access pathological proteins, e.g. amyloid-beta. A number of such antibody ligands have been developed, displaying differences in brain uptake, pharmacokinetics, and ability to bind and visualize the target in the brain of transgenic mice. Potential pathological changes related to neurodegeneration, e.g. misfolded proteins and neuroinflammation, are suggested as future targets for this novel type of radioligand. Challenges are also discussed, such as the temporal match of radionuclide half-life with the ligand’s pharmacokinetic profile and translation to human use. In conclusion, brain PET imaging using bispecific antibodies, modified for receptor-mediated transcytosis across the BBB, is a promising method for specifically visualizing molecules in the brain that are difficult to target with traditional small molecule ligands.

## Introduction

Positron emission tomography (PET) is a non-invasive, quantitative, functional imaging method. Clinically, PET is used to aid diagnosis, especially in cancer, where the radioactive glucose analogue [^18^F]FDG is used to localize primary tumours and metastases. PET has also become an important tool for diagnosis of brain disorders, since naturally it is difficult to obtain biosamples from the brain. Further, PET is an attractive method in translational research and drug development, as the same experiments can be performed in vivo in both animals and humans, and it allows for repeated investigations in one subject.

The main hurdle for the delivery of drugs (and radioligands) to the brain, irrespective of their size, is the blood–brain barrier (BBB), comprising tightly connected endothelial cells. Traditionally, PET radioligands for the central nervous system (CNS) have been based on small “drug-like” molecules preferably labelled with clinically compatible positron-emitting radionuclides such as carbon-11 (^11^C) or fluorine-18 (^18^F). Radioligands for brain imaging have to be fairly lipophilic to be able to pass through the BBB into the brain parenchyma. Unfortunately, increased lipophilicity also increases nonspecific distribution into the lipophilic brain tissue. This may lead to a poor specific-to-nonspecific PET signal. Further, and especially relevant in proteopathies such as Alzheimer’s disease (AD) and Parkinson’s disease (PD), it is unlikely that small-molecule radioligands could discriminate between different aggregation forms of a protein or proteins with similar fibrillary structures. Thus, in line with the shift in therapeutic focus from small-molecule drugs to biologics, antibodies or fragments thereof could turn out to be a completely novel class of neuroPET radioligands and could be used for highly specific PET imaging in the CNS, including imaging of target proteins for which radioligands are lacking today.

## Antibody transport across the blood–brain barrier

Radioligands based on antibodies or other proteins have already been introduced for peripheral targets related to cancer diagnostics and theranostics, including some applications in clinical use as well [1, 2]. However, antibodies are large molecules, displaying highly restrictive BBB transcytosis. It has been reported that only 0.1% of peripherally administered antibody reaches the brain [3, 4], and it has even been questioned whether antibodies penetrate the brain parenchyma at all, or whether antibody concentrations measured in the brain rather reflect transport from the blood into the cerebrospinal fluid (CSF) [5]. Thus, antibodies and other proteins will most likely have to be specifically engineered for facilitated transport across the BBB to enable their use as PET radioligands within the CNS.

**Fig. 1:**
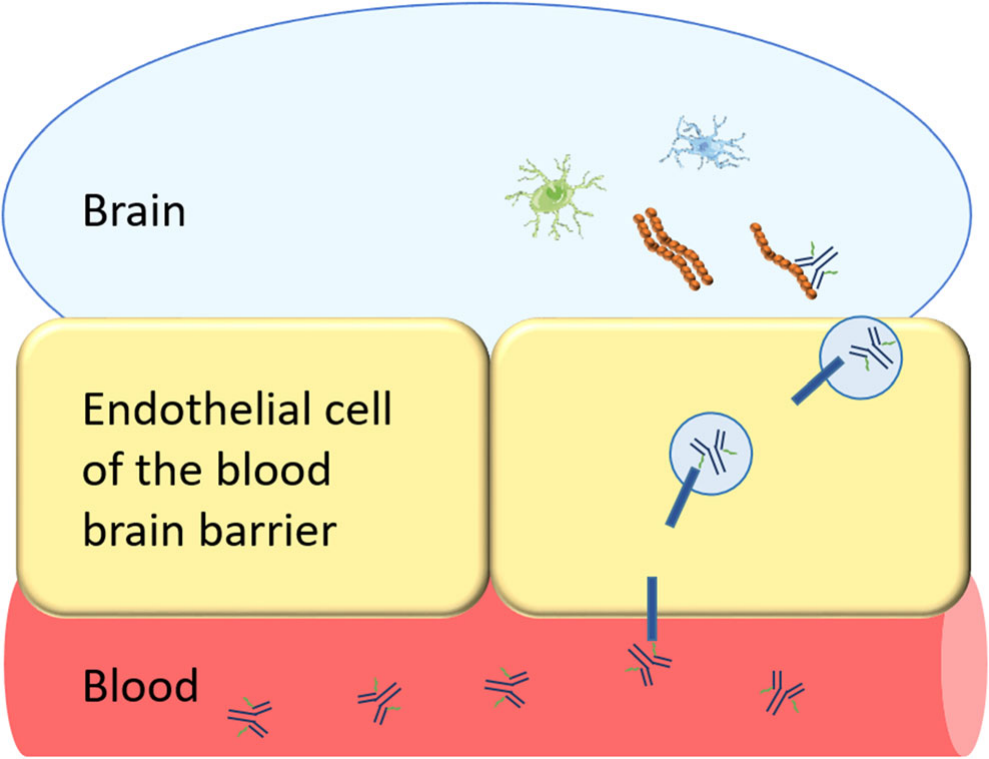
Transferrin receptor (TfR)-mediated transcytosis of a bispecific antibody. The bispecific antibody binds to TfR at the luminal side of the BBB. TfR transports the bispecific antibody inside an endosome across the BBB. The bispecific antibody is released on the abluminal side of the BBB and can then bind to its target (e.g. Aβ, reactive astrocytes, microglia) in the brain parenchyma

Carrier-mediated transporters at the BBB have been described for essential compounds such as glucose and amino acids, while insulin and transferrin (Tf) are transported into the brain with receptor-mediated transcytosis (RMT). Especially the transferrin receptor (TfR) has been used to increase transport of antibody-based therapeutics across the BBB. This has in recent years proven to be a successful strategy in several preclinical studies [6–8]. In early clinical studies, the insulin receptor was utilized for boosting the brain delivery of recombinant lysosomal enzyme α-L-iduronidase, aimed at treating patients with the metabolic disorder mucopolysaccharidosis (MPS) type I [9]. Other reported strategies for increasing brain delivery of antibodies include various binders targeting the endothelial cell surface protein CD98 [10] and the single-domain antibody fragment FC5, targeting the transmembrane protein 50A (TMEM50A) [11, 12]. The common feature of all the reported studies is that a binder for the selected BBB receptor (insulin receptor, TfR, CD98, etc.) is fused, either by recombinant expression or chemical conjugation, to the therapeutic antibody/protein. The generated bispecific protein or antibody is then expected to bind its BBB receptor and subsequently, after successful delivery across the BBB, interact with its primary intrabrain target (Fig. 1). Several initiatives are ongoing to find proteins expressed at the BBB that could act as brain shuttles for therapeutic antibodies or other biologics [13, 14]. This molecular “Trojan horse” strategy, in addition to enhancing the concentration of therapeutic biologics in the brain, can be applied to generate bispecific radioligands. This review will focus on TfR binding bispecific radioligands.

## The transferrin receptor

The TfR is found in two different isoforms: TfR1 and TfR2. TfR1 (also known as cluster of differentiation 71, or CD71) is expressed in brain endothelial cells, hepatocytes, and erythrocyte precursors, especially in bone marrow, lung, and other rapidly dividing cells. TfR2 is only expressed in hepatocytes, enterocytes of the small intestine, and erythroid cells. Both forms are expressed as transmembrane glycoproteins composed of two disulfide-linked monomers joined by two disulfide bonds. Each monomer binds one holo-transferrin molecule, creating an iron-Tf-TfR complex which enters the cell by endocytosis. In the endosome, the lower pH of around 5.5 will cause Tf to release its iron ions, which can subsequently be used by the cell. The TfR-Tf complex will then be recycled to the cell surface.

A number of antibodies have been generated against TfR1, among them the monoclonal mouse antibody OX-26 [15] that binds to the rat TfR1, and rat antibody 8D3 [16] that binds to the murine TfR1. Initially, OX-26 and 8D3 were developed for immunohistochemical visualisation of brain capillaries. However, it was later discovered that the antibodies were also found in high concentrations in the brain after in vivo systemic administration, and further, that when fused to a protein cargo, they were able to carry its cargo across the BBB [15, 17, 18].

despite binding to TfR1 [19]. It is debated which factors govern the ability to induce TfR1 transcytosis. Some studies have suggested that low/moderate affinity promotes transcytosis, while high affinity reduces release from the receptor and leads to lysosomal degradation [8]. Other studies have suggested that the binding valency to the TfR1 is of importance, monovalent binding being more efficient than bivalent binding, which may cause TfR clustering at the cell surface and intracellular degradation [6]. These theories are not necessarily conflicting, as monovalent binding in general results in lower affinity (avidity). Yet another feature that may govern transcytosis efficiency is pH-dependent affinity to the receptor. Ideally, the antibody should have moderate TfR affinity at neutral pH for optimal engagement at the cell surface, and low affinity at low pH for release in the acidic endosome and further transport across the cell, into the brain. While it has not been systematically studied and reported, the location of the TfR binding epitope may also be of importance for an antibody’s ability to undergo TfR-mediated transcytosis.

## Alzheimer’s disease and amyloid-β

**Fig. 2:**
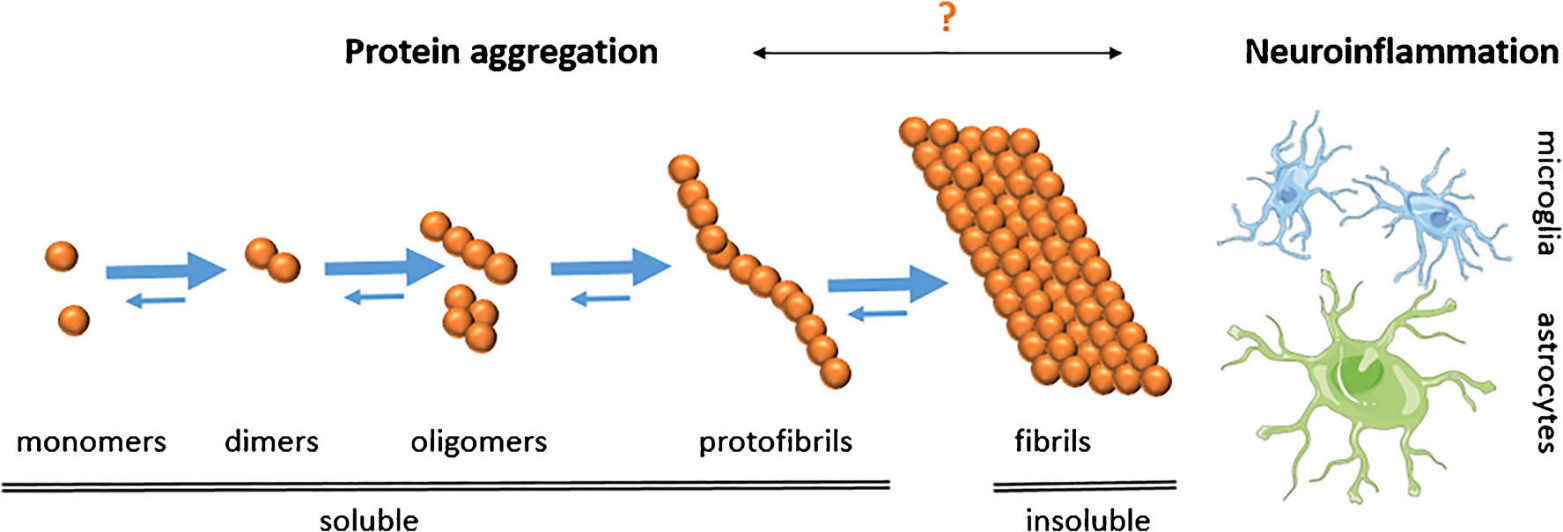
Protein misfolding causes aggregation of monomeric Aβ into oligomers and protofibrils, which ultimately form insoluble fibrils, i.e. the main constituent of Aβ plaques which are characteristic for AD. Of the soluble species, oligomers/protofibrils are most likely the toxic forms of Aβ responsible for neurodegeneration. However, all amyloid PET ligands today detect fibrillar Aβ. The trigger for neuroinflammation is debated, but both activated microglia and reactive astrocytes are observed in the AD brain

Insoluble plaques of amyloid-beta (Aβ) in the brain of AD patients can today be visualized with PET using the thioflavin-T-derived small-molecule radioligand [^11^C]PIB (Pittsburgh compound B) that was developed by the Uppsala University Hospital PET Centre in collaboration with Pittsburgh University in the early 2000s [20]. The introduction of amyloid imaging has been an important improvement in clinical diagnosis of AD, especially in cases when the cause of dementia is unclear [21]. However, [^11^C]PIB, and all later developed analogues, bind to the beta-sheet structure of insoluble plaques and give rise to a fairly static signal that does not correlate well with disease severity during the clinical stages of AD [22, 23]. Further, since [^11^C]PIB does not bind to diffuse plaques [24], a form of Aβ deposits that dominates AD pathology in about 5% of AD patients, there is a substantial risk for a false-negative diagnosis with [^11^C]PIB. In addition, it appears that soluble aggregates of Aβ, e.g. protofibrils/oligomers formed before fibrils (Fig. 2), are involved in the pathological process that leads to neuronal degeneration, and further, that levels of soluble aggregates correlate better than insoluble plaques with disease severity [25–27]. Thus, efforts today are devoted towards therapeutic targeting of soluble Aβ, including protofibrils/oligomers [28], and the development of PET ligands that can visualize these Aβ species is therefore also of great interest.

## Antibody-based radioligands for imaging of Aβ

**Fig. 3:**
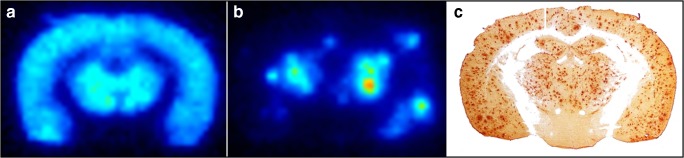
Aβ pathology visualized in the transgenic (tg-ArcSwe) mouse brain. Ex vivo autoradiography brain sections isolated 6 days after intravenous administration of (**a**) a bispecific TfR1- and Aβ-binding antibody or (**b**) an unmodified Aβ-binding antibody. The spatial distribution of the bispecific antibody corresponds well with the Aβ40 immunohistochemistry shown in (**c**), while the non-modified antibody is accumulated centrally around the ventricles and in high-intensity deposits

One attractive feature of antibodies is that they can be generated to be specific, or selective, for certain aggregation forms or modifications (truncation, phosphorylation, etc.) of a protein. A few attempts to develop radioligands based on antibodies, mainly targeting Aβ, without a specific BBB shuttle moiety have been described in the literature, although some have been conjugated to nonspecific BBB modifiers like polyethylene glycol (PEG) [29–33]. While few of the published articles show PET images or whole brain section ex vivo autoradiography, most of them report elevated brain concentrations of Aβ antibodies in transgenic versus wild-type (WT) mice. However, the total brain concentration of radiolabelled antibodies is low or animals are not perfused to exclude the radioactivity in blood, which makes it difficult to determine antibody concentrations in the brain parenchyma [30–32]. Fissers and co-workers include images of ^89^Zr-labelled JRF/AβN/25, a monoclonal antibody directed against full-length Aβ [29]. The radioligand showed in vitro stability and high affinity to Aβ, but ex vivo autoradiography showed that the radioligand mainly accumulated in local high-intensity deposits. This binding pattern has also been observed for other Aβ-binding antibodies such as 3D6 and mAb158 [7, 34]. It is likely that the high-intensity deposits represent radioligand binding to Aβ aggregates in the brain vasculature, i.e. cerebral amyloid angiopathy (CAA). To enable PET imaging of intrabrain Aβ, it is important that the radioligand is able to access the whole brain parenchyma, as illustrated in Fig. 3. This is most efficiently achieved by transport across the BBB in the whole brain capillary network, since diffusion from potential CAA deposits or areas of broken BBB into the deeper brain parenchyma is likely to be too slow [35, 36].

**Fig. 4:**
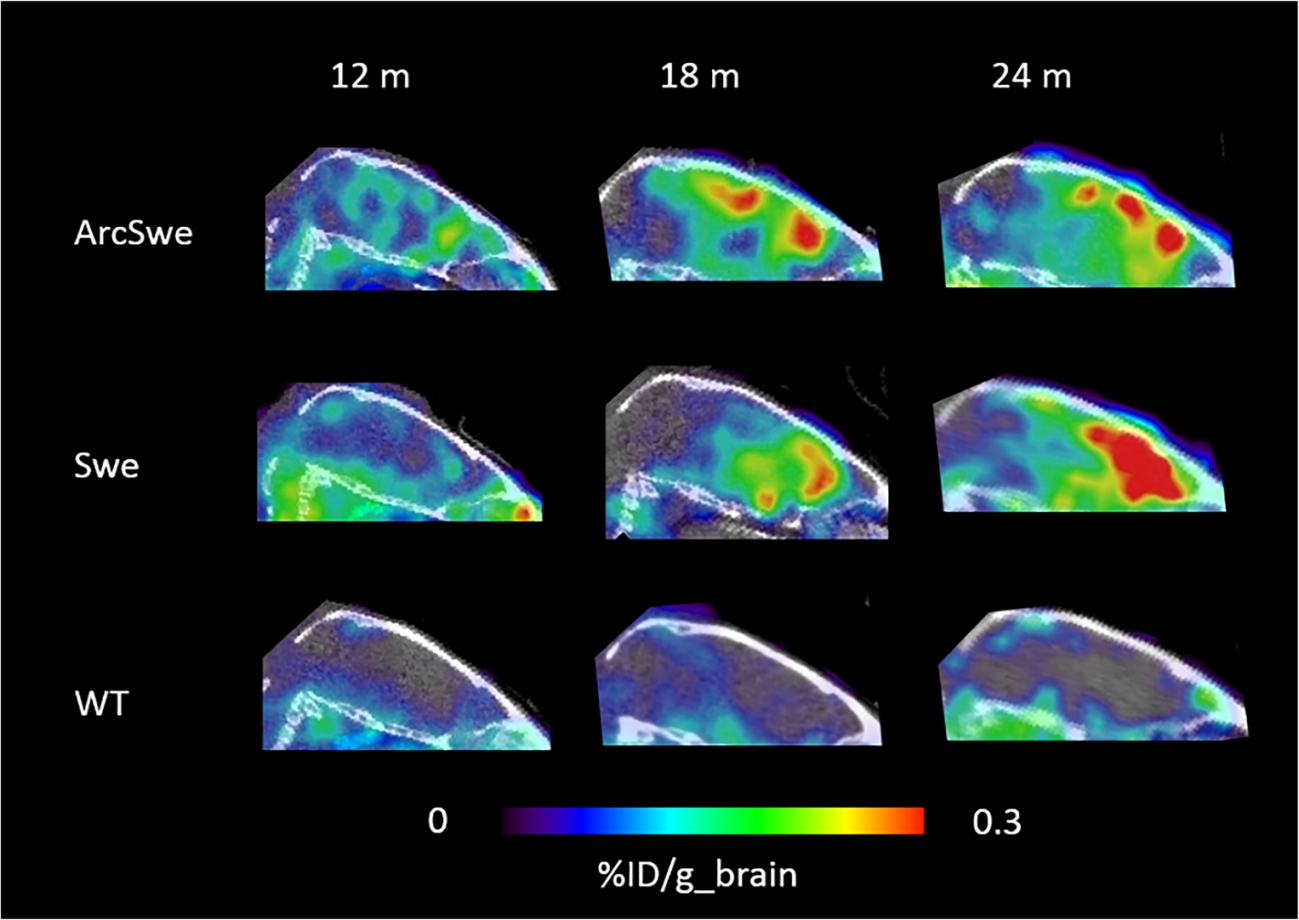
Sagittal PET images obtained at 3 days after administration of the bispecific radioligand [^124^I]8D3-F(ab’)_2_-h158 in two mouse models of AD (ArcSwe and Swe) and wild-type (WT) mice at different ages

**Fig. 5:**
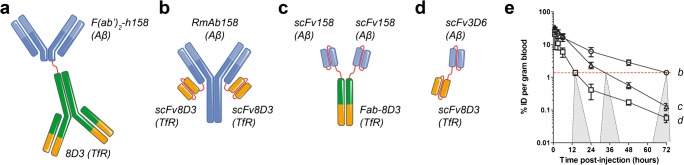
Different formats of bispecific, brain-penetrating antibodies used for PET imaging of Aβ pathology in AD transgenic mouse models. **a** F(ab’)_2_ fragment of humanized Aβ protofibril-selective mAb158, chemically coupled to full 8D3 antibody (Mw ~270 kDa). **b** Recombinant variant of mAb158 (RmAb158) with scFv8D3 recombinantly fused to the C terminus of each of the light chains (Mw ~210 kDa). **c** Tribody composed of two scFv158 attached to each chain of a Fab-8D3, brought together by the natural combination of the Fab fragment (Mw ~110 kDa). **d** Tandem-scFv composed of scFv3D6 fused via a polypeptide linker to scFv8D3 (Mw ~58 kDa). **e** Blood elimination curves of recombinant antibody ligands (**b**), (**c**), and (**d**). The dashed red line represents an approximate antibody blood concentration threshold below which PET imaging is feasible. From the intersection of each antibody’s blood curve with the threshold line, there is a projection to a time window where AD transgenic mice can at the earliest be discriminated from wild-type mice

The first bispecific antibody-based PET study that took advantage of TfR1 as a brain shuttle utilized the complete 8D3 antibody chemically fused to a F(ab’)_2_ fragment of an Aβ protofibril-binding antibody, mAb158 (Fig. 5a). Hence, the TfR1 binding was bivalent and led to a 15-fold increase in brain concentrations, measured 4 h after administration, compared with the F(ab’)_2_ fragment of the original antibody [37]. The same study also showed that 8D3 was equally efficient in shuttling a F(ab’)_2_ fragment of an antibody without any intrabrain target, and that the BBB transport took place both in WT animals and in two different models of AD. Thus, the study showed that BBB transcytosis per se was not influenced by subsequent binding (or no binding) in the brain parenchyma. The bispecific antibody was radiolabelled with ^124^I, and PET images obtained 3 days post-administration, when unbound radioligand had been cleared, showed an increasing signal intensity with age (i.e. with increasing Aβ pathology) in the two AD animal models, while brains of WT mice were devoid of signal regardless of age (Fig. 4) [37, 38].

To introduce monovalent TfR binding, fragments of 8D3 have been recombinantly fused with the primary antibody (Fig. 5). RmAb158-scFv8D3 (Fig. 5b) resembles the original full-sized mAb158 with an intact Fc domain [39], with two scFv8D3 fragments attached to each of the IgG light chains. The short linkers between scFv8D3 and the light chain hinder simultaneous binding to TfR1 with both scFv8D3, while the two copies of scFv8D3 increase the chance of binding. Further, since RmAb158-scFv8D3 is a symmetrical antibody, it is easier to produce than asymmetrical, bispecific full-sized IgG-like antibodies that have been described in the literature [6, 8]. PET imaging showed that [^124^I]RmAb158-scfv8D3 reached considerably higher brain concentrations, resulting in higher-contrast images compared with [^124^I]8D3-F(ab’)_2_-h158 (Fig. 5a), which binds bivalently to TfR [39]. In subsequent studies, smaller bispecific constructs have been used to promote faster clearance from blood, i.e. a feature that is desired for PET radioligands to minimize the radioactivity in the vascular volume of the brain. These studies showed that a tribody, based on two scFv158 and a Fab-8D3 [40], with a size of approximately 110 kDa (Fig. 5c), and a tandem-scFv based on an scFv3D6 fused to an scFv8D3 [41], with a size of approximately 58 kDa (Fig. 5d), were more rapidly cleared from blood than the larger 210 kDa [^124^I]RmAb158-scfv8D3, and allowed for imaging of Aβ pathology at an earlier time point after administration (Fig. 5e).

### Antibody vs PIB PET

**Fig. 6:**
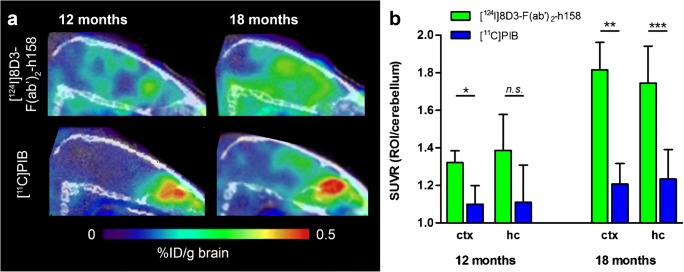
Comparison of antibody and PIB PET imaging. **a** Sagittal view of PET images obtained from 12- and 18-month-old tg-ArcSwe mice 3 days post-injection of [^124^I]8D3-F(ab’)_2_-h158 (upper panel) in comparison with mice scanned 40–60 min after injection of [^11^C]PIB (lower panel). **b** Quantification of PET images from (**a**) expressed as standardized uptake value ratio (SUVR) of two regions of interest, cortex (ctx) and hippocampus (hc), using cerebellum as reference region. Significant differences were observed for all regions and ages except for hippocampus in the 12-month-old animals

PET imaging with bispecific antibodies has been performed primarily in two AD mouse models, tg-ArcSwe and tg-Swe [37, 38, 41], expressing human APP with both the Arctic (E693G) and Swedish (KM670/671NL) amyloid precursor protein (APP) mutations or the Swedish mutation alone. Tg-ArcSwe is characterized by early formation of soluble Aβ aggregates and subsequent formation of dense core plaques, closely mimicking plaques formed in the human brain, while tg-Swe has a late-onset pathology, rapid progression, and less dense plaques [42]. PET imaging with [^124^I]8D3-F(ab’)_2_-h158 and [^11^C]PIB revealed an intense PET signal with the antibody-based ligand, but a fairly low signal with [^11^C]PIB, which was readily detectable only in aged mice (18 months) [37] (Fig. 6a). When quantified as standardized uptake value ratio (SUVR), using cerebellum as reference region, [^124^I]8D3-F(ab’)_2_-h158 showed earlier and better discrimination between transgenic and WT mice compared with [^11^C]PIB (Fig. 6b) [37, 41]. Interestingly, tg-ArcSwe mice, showing more dense plaque pathology, had higher [^11^C]PIB retention than Swe mice, which instead gave a higher signal with the antibody ligand. This is in line with the two ligands’ binding characteristics, i.e. [^11^C]PIB requires dense core plaques, while [^124^I]8D3-F(ab’)_2_-h158 binds to diffuse assemblies including oligomers and protofibrils. Quantification of Aβ levels in brain tissue from the previously scanned animals showed that the [^124^I]8D3-F(ab’)_2_-h158 PET signal correlated closely with protofibril levels, while no correlation was observed with total Aβ levels (corresponding to plaque load). The need for dense core plaques for imaging of Aβ with [^11^C]PIB and analogues has also been shown in other studies. Snellman and co-workers demonstrated increasing [^11^C]PIB signal with age in APP23, a model with compact Aβ assemblies, while no such trend and very low brain concentrations were found in Tg2576 mice, a model expressing only the Swedish mutation and thus characterized by the absence of dense plaques [43]. Brendel and co-workers conducted a cross-sectional study with [^18^F]florbetaben in four different AD mouse models and showed large variation in radioligand accumulation between the different models, including low accumulation in two models based on the Swedish mutation: APPSwe and APP/PS1dE9 [44]. Only one model, PS2APP, showed SUVRs above 1.1 at the age of 15 months. Further, a clinical study demonstrated that AD patients with mainly diffuse pathology were [^11^C]PIB negative [24]. Taken together this implies that antibody-based PET, but not [^11^C]PIB, is able to visualize and quantify early formed and diffuse Aβ assemblies.

Additional evidence for the sensitivity of antibody PET is provided by a study where 10-month-old tg-ArcSwe mice were treated for 3 months with a BACE-1 inhibitor to decrease Aβ production. PET imaging with the recombinant [^124^I]RmAb158-scfv8D3 readily detected reduced Aβ levels in treated compared to non-treated animals, at an age when [^11^C]PIB can hardly detect Aβ [45].

## Other pathological changes in need of novel radioligands

### α-Synuclein

Similar to AD, protein misfolding and aggregation is also a pathological feature in PD. In PD, the presynaptic protein α-syn initially forms oligomers and later insoluble aggregates. There are presently no PET ligands available for imaging of either soluble or insoluble α-syn, and several research programmes are aimed at developing small-molecule PET ligands for α-syn [46]. The lack of in vivo biomarkers for α-syn is a major limitation for the development of disease-modifying treatments for PD, and the Michael J. Fox Foundation (MJFF) recently announced a $2 million prize to the first team to develop a viable selective α-syn PET radioligand. A number of initiatives to develop a radioligand for α-syn are ongoing, and most of these are based on small molecules. Although some promising compounds have been presented, one major hurdle is the cross-reactivity and binding to Aβ. Even if the affinity of a specific compound is much higher for α-syn than Aβ, the abundance and availability of Aβ may be higher. Thus, it might be difficult to differentiate between an α-syn-containing brain and an Aβ-containing brain, even if Aβ levels are low. This may be especially a problem when different protein pathologies coexist, which is often the case in older individuals. In this respect, highly specific antibodies for α-syn may be a new option for developing radioligands truly specific for α-syn. One potential challenge is that the majority of aggregated α-syn in the brain appears to be intracellular, and thus an additional barrier has to be conquered by the bispecific antibody in order to reach its primary target.

### Microglia and astrocytes

PET imaging of activated microglia and reactive astrocytes has been used as an indication of neuroinflammation. For example, a number of PET radioligands have been developed for the 18-kDa translocator protein (TSPO), which is highly expressed on activated microglia. However, quantitative interpretation of the PET signal with the second- and third-generation TSPO PET radioligands is confounded by large interindividual variability in binding affinity due to a genetic polymorphism leading to a trimodal distribution, reflecting high-affinity binders (HABs), low-affinity binders (LABs), and mixed-affinity binders (MABs) [47]. In addition, TSPO is also expressed on astrocytes, and hence the TSPO ligands are not specific for microglia. Reactive astrocytes, also indicative of neuroinflammation, can be imaged with the PET radioligand deuterium-L-depreneyl ([^11^C]DED). [^11^C]DED binds to monoamine oxidase-B, primarily found in activated astrocytes, and although studies indicate that the radioligand indeed visualizes astrocytosis, its binding differs from that of other astrocytic markers often used in immunohistochemical analysis in post-mortem neuropathological studies, such as glial fibrillary acidic protein (GFAP) [48, 49]. Attempts have been made to image GFAP with antibody fragments [50], and myriad well-characterized antibodies for proteins expressed on microglia and astrocytes have been described in the literature. Hence, the possibility of engineering these into bispecific brain-penetrating radioligands is tempting and could potentially allow for “in vivo immunohistochemistry” of classical ex vivo immunohistochemical targets.

## Challenges

### TfR transport capacity

One central paradigm of PET is the use of tracer doses that do not elicit a pharmacological response or occupy a significant fraction of potential binding sites. The use of doses above true tracer doses may have an impact on the PET signal, and hence the interpretation of the study. It is therefore important to estimate the capacity of the TfR. For example, a study using [^125^I]RmAb158-scfv8D3 showed that, compared with unmodified [^125^I]RmAb158, the transport of bispecific radioligand into the brain was increased almost 100-fold at tracer doses (0.05 mg/kg), while a tenfold increase was observed at a dose of 10 mg/kg [39]. The blood pharmacokinetics were linear, i.e. the half-life in blood was the same for the tracer and the pharmacological dose, and did therefore not contribute to the changed BBB transcytosis efficacy. Moreover, the study showed that co-administration of full-sized 8D3, also at a dose of 10 mg/kg, even more efficiently inhibited transcytosis by reducing the brain uptake of [^125^I]RmAb158-scfv8D3 to threefold more than [^125^I]RmAb158. The lower inhibition capacities of RmAb158-scfv8D3 compared with 8D3 can most likely be explained by the former compound’s monovalent TfR binding.

**Fig. 7:**
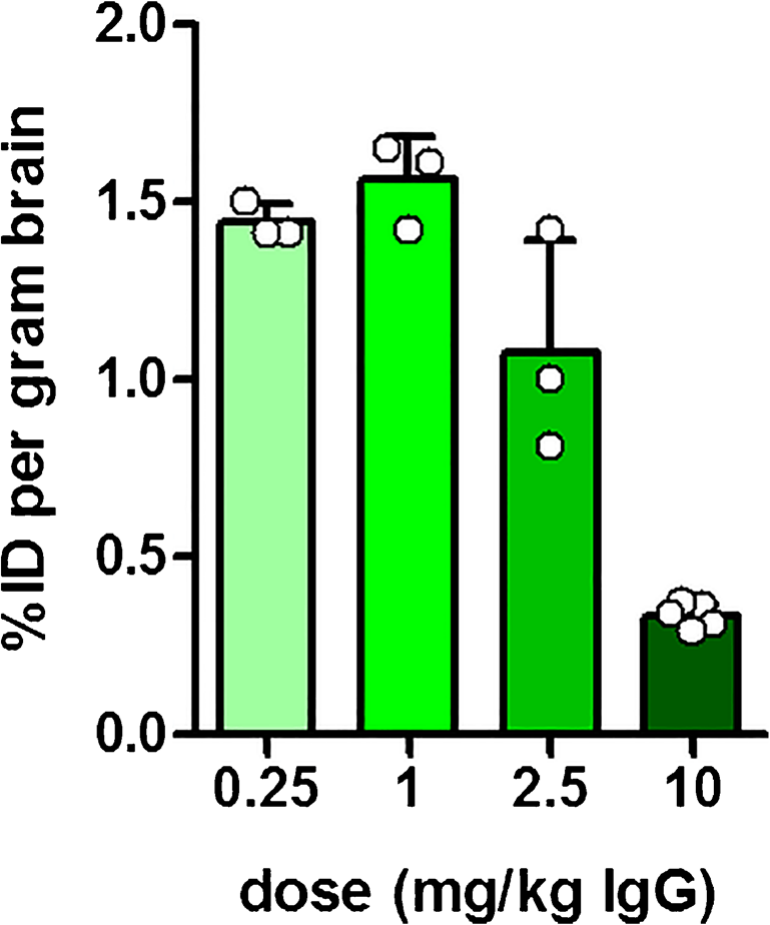
Dose vs brain uptake of RmAb158-scFv8D3. Brain antibody uptake (% of injected dose per gram brain tissue) was measured 2 h post-injection of RmAb158-scFv8D3 at doses ranging from 0.25 mg/kg body weight to a high therapeutic dose of 10 mg/kg body weight. While antibody doses relevant to PET imaging (below 0.5 mg/kg) had no impact on brain delivery, doses above 1 mg/kg seemed to saturate the TfR transport mechanism, resulting in reduced brain uptake

To further understand the TfR transcytosis efficiency in relation to TfR occupation, brain uptake was investigated at different intravenously administered doses of RmAb158-scfv8D3. Doses up to 1 mg/kg had no impact on BBB transport, resulting in brain antibody concentrations of around 1.5% of the injected dose per gram brain tissue at 2 h post-injection. At higher doses, TfR seemed to be saturated, and the brain uptake was reduced in a dose-dependent manner (Fig. 7). While this may be a concern for therapeutic applications, all PET studies described here were conducted at antibody doses below 0.5 mg/kg body weight, calculated as IgG equivalents, i.e. below the limit of TfR saturation.

### Radiolabelling

Protein- and antibody-based PET radioligands have traditionally been radiolabelled with radionuclides such as iodine-124 (^124^I; half-life 4.2 days), zirconium-89 (^89^Zr; half-life 3.3 days), or gallium-68 (^68^Ga; half-life 68 min). Except for ^124^I, these radionuclides require the attachment of a chelator on the antibody/protein backbone before the introduction of the radionuclide. The instability of the chelators was initially a major challenge, but new and more stable versions that may be better suited for clinical use have been introduced [51]. On the other hand, ^124^I has not been a preferred alternative due to its accumulation in the thyroid, resulting in high local exposure. Further, the above-mentioned radionuclides are not ideal for clinical use due to low fraction of positron decay (26% for ^124^I and 23% for ^89^Zr—only this fraction of radioactivity is detected by PET). Also, the high energy of ^124^I positrons, which allows them to travel a longer distance in the tissue before electron annihilation, causes low-resolution PET images. In contrast, the low-energy positrons from ^18^F decay generate PET images with high resolution.

For small-molecule radioligands used clinically, fluorine-18 (^18^F; half-life 110 min) is the first choice for radiolabelling. However, radiochemistry methods for introducing ^18^F on antibody-based ligands have been lacking. With the increased interest in protein-based radioligands, increasing efforts have also been observed when it comes to ^18^F radiolabelling. Different strategies involving” click-chemistry”, e.g. Diels-Alder reaction, have been described [52]; the radiolabelling is somewhat similar to the above-mentioned chelator methods, i.e. a two-step process. First, the antibody/protein is modified by introducing a small-molecule group that in a second step can react with another small molecule that carries the ^18^F. Some methods for direct radiolabelling of amino acids, e.g. amine groups, with ^18^F have also been described [53, 54].

### Pharmacokinetics

The radioactivity measured with PET in the brain (or any tissue of interest) comprises radioactivity in the tissue itself as well as radioactivity in the blood pool of the tissue. For example, around 3% of the brain volume is blood [55, 56]. Thus, PET radioligands should be cleared from the blood fairly rapidly to minimize the radioactivity contribution from the blood pool of the tissue. This is especially important for the brain and for neuroPET radioligands with limited brain distribution. Full-sized antibodies are generally associated with long systemic half-life, while a more rapid elimination can be achieved by modifying antibodies into fragments (Fab, F(ab’)_2_, scFv). However, even fragments may display half-lives that are not compatible with clinically preferred radionuclides ^11^C and ^18^F. In addition, specific and nonspecific binding to peripheral targets may contribute to the observed half-life. For example, binding to soluble circulating and erythrocyte-expressed TfR1 may either prolong or shorten the circulation time. Although there are limited published data, it appears that smaller size and lower TfR1 affinity leads to faster elimination from blood [40, 41]. Hence, studying the systemic pharmacokinetics of antibody-based ligands is essential before deciding on a labelling strategy. Moreover, to achieve a high signal-to-noise ratio, unbound ligand must also be eliminated from the brain within a time frame that matches the half-life of the radionuclide. Knowledge about clearance rates and routes of bispecific antibodies from the brain is sparse, and more research will be required to elucidate whether brain clearance is passive or mediated by active transport mechanisms, and whether it is size-dependent.

### Translation

Translation from preclinical imaging to clinical imaging is challenging from many perspectives. In addition to dosimetry and pharmacokinetics that may differ between different species, the actual target under investigation may also be different although its biological “purpose” may be the same in different species. In PET, species differences have been observed for both neuroreceptors and transporters at the BBB [57, 58]. The differences include different density of the target, different function and different amino acid composition. Limited knowledge about species-related differences in BBB receptors that mediate transcytosis, e.g. the TfR1 and the insulin receptor, makes it difficult to predict the brain delivery in humans based on preclinical data. The murine TfR1 and the human TfR1 differ in the amino acid sequence at the domain where the 8D3 antibody binds, resulting in no binding of 8D3 to the human TfR. However, antibodies binding human TfR1 at the same domain as 8D3 have been generated and shown to successfully shuttle biologics across the in vitro human BBB and in vivo in monkeys [59, 60]. Attempts to generate species-independent TfR1 antibodies, which would aid in translation, have been described, but with limited success. One exception is perhaps the generation of variable new antigen receptors (VNARs) that appear to bind both mTfR1 and hTfR1 [61]. Published data on the capacity of the VNARs to shuttle antibody cargos across the BBB is limited, however, and it appears that the VNARS may be less efficient for BBB transport of full-sized antibodies compared with smaller fragments. This is not the case for 8D3. In summary, it appears that TfR1 can be used in both mice and men, but may require species-specific TfR1 binders. The expression of TfR1 at the murine and human BBB has been reported to be similar [5].

Very little data on translatability and species differences have been reported for CD98 and the TMEM50A binder FC5, although they are claimed to be species-independent [10–12].

## Discussion

The development of antibody-based PET ligands for brain disorders has so far not been feasible, as techniques to facilitate large protein delivery to the brain have been lacking. Still, antibodies have many benefits, particularly their ability to bind specifically to their target, which is advantageous for obtaining PET images without background noise caused by nonspecific binding. Thus, the development of protein engineering strategies to increase antibody concentrations in the brain may enable a completely new class of radioligands with no or very low nonspecific binding.

The high affinity and specificity of antibodies are attractive, especially when specific aggregation forms of a protein are of interest. This is the case for many misfolded proteins, e.g. Aβ and α-syn, for which protein aggregates representing intermediate stages in the aggregation pathway is believed to be more toxic and dynamic than the insoluble end state deposits. In addition, numerous antibodies have been described for inflammation markers on astrocytes and microglia. Generation of bispecific antibodies able to pass the BBB based on these well-characterized and frequently used antibodies for immunohistochemical analyses could likely lead to novel in vivo imaging biomarkers.

Although already shown to be successful in the preclinical setting, the translation into clinical use will require the development of new radiochemistry for incorporation of more clinically suitable radionuclides such as ^18^F. A number of radiochemical strategies have been described, but large-scale synthesis and reproducibility has to be improved. However, labelling antibodies with ^18^F, which has a half-life of less than 2 h, will also require fast clearance of the ligand from blood and of unbound ligand from the brain. Further research on the pharmacokinetics of bispecific antibody ligands of different formats is needed to optimize these parameters for clinical development.

Another challenge is finding species-independent BBB shuttles, or validated shuttles in higher species. This has to some extent been accomplished for TfR1, although no species-independent binders have been proven to be as efficient as current mTfR1 binders. Several large-scale projects aiming to discover novel shuttles beyond TfR binders are ongoing.

In conclusion, we have already entered the era of biologics, both for the periphery and for the CNS, and it is likely that antibody- or protein-based radioligands will also become an important class of PET radioligands. A number of preclinical studies have shown the feasibility of antibody-based imaging of Aβ pathology in the brain, and although some hurdles remain, this novel class of tracers is likely to enter clinical development within the next few years.
